# Dynamics of Quantum Networks in Noisy Environments

**DOI:** 10.3390/e25010157

**Published:** 2023-01-12

**Authors:** Chang-Yue Zhang, Zhu-Jun Zheng, Shao-Ming Fei, Mang Feng

**Affiliations:** 1 Department of Mathematics, South China University of Technology, Guangzhou 510641, China; 2 School of Mathematical Sciences, Capital Normal University, Beijing 100048, China; 3 Max-Planck-Institute for Mathematics in the Sciences, 04103 Leipzig, Germany; 4 State Key Laboratory of Magnetic Resonance and Atomic and Molecular Physics, Wuhan Institute of Physics and Mathematics Innovation Academy of Precision Measurement Science and Technology, Chinese Academy of Sciences, Wuhan 430071, China; 5 Research Center for Quantum Precision Measurement, Institute of Industry Technology, Guangzhou and Chinese Academy of Sciences, Guangzhou 511458, China

**Keywords:** quantum networks, quantum noise, evolution time

## Abstract

Noise exists inherently in realistic quantum systems and affects the evolution of quantum systems. We investigate the dynamics of quantum networks in noisy environments by using the fidelity of the quantum evolved states and the classical percolation theory. We propose an analytical framework that allows us to characterize the stability of quantum networks in terms of quantum noises and network topologies. The calculation results of the framework determine the maximal time that quantum networks with different network topologies can maintain the ability to communicate under noise. We demonstrate the results of the framework through examples of specific graphs under amplitude damping and phase damping noises. We further consider the capacity of the quantum network in a noisy environment according to the proposed framework. The analytical framework helps us better understand the evolution time of a quantum network and provides a reference for designing large quantum networks.

## 1. Introduction

Quantum networks play significant roles in quantum computation and quantum communication, such as quantum key distribution (QKD) [1,2,3,4], which has been experimentally demonstrated based on the DARPA quantum network [5], the Tokyo QKD network [6] and the Chinese satellite quantum network [7], and has become a research hotspot in secure and efficient information transmission.

Due to the magic quantum properties [8], the dynamics of quantum networks is fundamentally different from that of traditional networks, which involves the effect that the quantum network structures change with the evolution of the quantum states [9,10]. It is well known that with only the use of maximally entangled quantum states, one can achieve perfect quantum communication in point-to-point protocols, such as quantum teleportation [11]. Therefore, Acín et al. [12] proposed the concept of entanglement percolation, aiming to establish a maximally entangled state between two arbitrary neighboring nodes of a quantum network. More interestingly, they combined the problem of establishing the maximally entangled states between nodes with the classical percolation in statistical mechanics [13] and demonstrated that the phase transitions are very useful in optimizing quantum networks. Later, the research on entanglement percolation in quantum networks also attracted much attention [14,15,16,17].

In reality, a quantum system will unexpectedly interact with outside environments. These unexpected interactions are always considered noise [8]. In previous works, the effect of noisy environments on quantum teleportation [18,19,20,21,22], quantum key distribution [23,24,25] and remote state preparation [26,27] have been investigated. However, less is studied on the problem of how quantum networks evolve in noisy environments. As the effect of noise on a quantum network is essential, in order to discuss this question, we propose an analytical framework to study the duration of quantum networks in noisy environments based on the classic percolation in statistical mechanics [13].

In the analytical framework, we use percolation theory to study the dynamics of noisy quantum networks, which has not been applied to the study of noisy systems in similar configurations. Our approach applies to different quantum network models; that is, if one considers different quantum networks, the maximum times of the evolution of different quantum networks can be calculated. The proposed framework is a useful analytical tool for the design of a practical quantum network. Analyzing the dynamics of the quantum networks allows us to determine the initial conditions for improving the performance of quantum communication in noisy environments. Actually, our analytical framework models the complex dynamics of a quantum network as bond percolation by using fidelity as a bridge. We can characterize the dynamics of the quantum network adopting the approach, which only needs to consider the evolution of a corresponding initial link under noise. Quantum network structures can be optimized to maintain quantum communication capabilities for a long time in a realistic physical context, based on the analysis results. The proper time interval of entanglement distribution can be set to save quantum resources via the calculating results from the analytical framework. Furthermore, quantum networks and their optimization is of explicit importance to emerging technologies, such as edge computing [28], and the presented percolation model can be useful when simulating an edge-computing scenario.

We consider the evolution time *t* for an arbitrary edge according to a quantum state in a quantum network and give an analytic expression for the fidelity of the evolved state in noisy environments. Subsequently, we associate fidelity with the probability of edge occupation. By combining the classical bond percolation threshold, we establish an expression to calculate the critical value of the time for a quantum network. In a noisy quantum network, there exists a critical value of evolution time, $t_{c}$, such that a giant connected component (GCC) appears if and only if $t<t_{c}$. Namely, the probability that two distant nodes can be connected by a path is distance-independent when $t<t_{c}$. If $t\geq t_{c}$, the probability decays exponentially with the length of the path between communication parties. Here, $t_{c}$ could be regarded as the maximal time that a quantum network under a noisy environment can maintain good communication between two arbitrary nodes.

In addition, we demonstrate the results of the framework through different quantum network topologies, including networks whose nodes are spatially distributed in a regular way according to geometries and the complex quantum networks whose nodes are randomly distributed. We further consider a decoherence model in a nuclear magnetic resonance (NMR) system whose spin is affected by phase damping and amplitude damping noises and calculate the value of time when the critical phenomenon occurs in this case. The calculation results exhibit the maximal time of different quantum network topologies staying stable. Finally, we investigate how the dynamics of quantum state affects the capacity of quantum networks in noisy environments and give an upper bound on the capacity of the quantum network.

## 2. Preliminaries

### 2.1. Quantum Network Model

A classical network consists of both nodes and edges that connect pairs of nodes. A node may be connected with other nodes by many edges, and the number of edges connected to a given node is called the degree of the node. A connected component or cluster is a subgraph of a network in which two arbitrary nodes are connected by at least one edge.

The quantum networks are also composed of nodes and edges, where the nodes can send and receive quantum states while the edges are quantum entangled states shared by the nodes. Mathematically, a quantum network is represented by a graph G(V,E) [12,14], where V={1,2,…,N} is the set of nodes, and E⊂V×V is the set of edges in the graph. An edge existing between two nodes represents the two nodes share at least one quantum state.

In the quantum network described above, any two nodes (communication parties) achieve a round of quantum communication of four phases. Phase (1) is responsible for generating initial links. Quantum nodes distributing quantum entanglement refer to network topology. Every two adjacent nodes share at least one entangled state, which can be used for quantum communication. In phase (2), paths between communication parties are found through a routing algorithm running on all nodes. Phase (3) is the process of establishing end-to-end links shared by communication parties. Intermediate nodes in the path perform entanglement swapping to build a new entanglement connecting communication parties [29,30,31,32]. In phase (4), communication parties use end-to-end links to complete information transmission.

**Figure 1:**
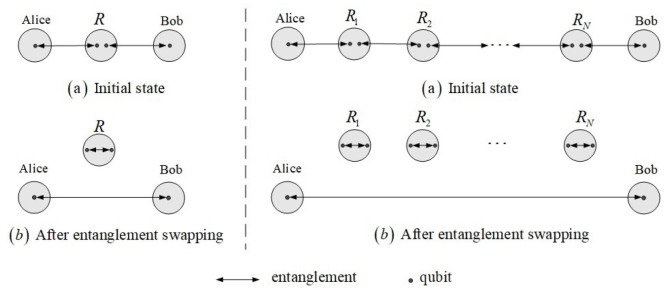
Entanglement swapping in the quantum network. Alice and Bob can establish a quantum channel through two steps. Step (a) is selecting a path from a quantum network, such as A→R→B or $A\to R_{1}\to R_{2}\to \cdots \to R_{N}\to B$. Step (b) is that intermediate nodes perform Bell state measurements to build a long-distance entanglement between the communication parties.

As shown in Figure 1, the goal of the two nodes (Alice and Bob) is to establish an entangled state for quantum communication. According to the four phases, this can be easily achieved in an ideal environment since a path between any two nodes always exists. In contrast, a practical quantum network is always affected by the environment. The evolution of quantum states under noise leads the dynamic of the quantum network to be more complex. The path between any two nodes does not always exist in the quantum network under noise. Therefore, we develop an analytical framework to quantify the stability of the quantum network in a noisy environment by analyzing the evolution of the initial link.

### 2.2. Percolation Model

**Figure 2:**
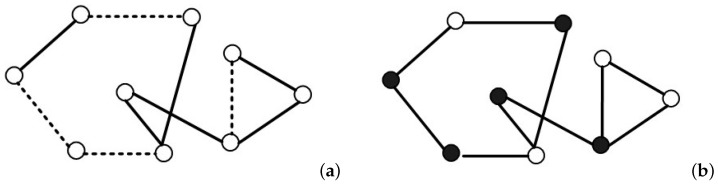
Schematic diagrams of bond percolation and site percolation. (**a**) Bond percolation; (**b**) Site percolation.

Percolation theory is a cornerstone of the theory of disordered media [13], which has been frequently employed to characterize the performance of network systems [33]. For the classical network, there are two main types of percolation: the bond percolation model and site percolation model (see Figure 2).

As shown in plot (a), the process of bond percolation considers that each edge is occupied with probability *p* (solid line) and unoccupied with probability 1−p (dotted line). If there is an edge between two arbitrary nodes, and the edge is connected (occupied) successfully with the probability *p*, one can discuss the distribution of the connected component size. For example, plot (a) contains two connected components with sizes of 2 and 6, respectively. In a large network, when the value of *p* is very small, a small number of clusters will appear in the network. With the increase in the probability *p*, clusters begin to grow and to be connected with each other. A GCC appears when *p* reaches a critical value $p_{c}$, which is the only component with the relative size finite (nonzero) in the large network limit. The network, in this case, can give rise to better information transmission. Here, $p_{c}$ is called the bond percolation threshold of the network.

The site percolation is defined by declaring each site is occupied with probability *q* (filled circle) and unoccupied otherwise (empty circle). As shown in the schematic diagram of site percolation, plot (b), there are three connected components with sizes 1, 2, and 2. Similarly, there exists a critical value of *q*, where the infinite cluster appears.

More general percolation problems can be formulated by the mixed percolation, whose probability is calculated using random elimination of sites and bonds simultaneously. More detailed introductions are available in Refs. [13,33,34,35,36].

## 3. The Evolution of Quantum Network

### 3.1. Analytical Framework

In the quantum domain, we propose a framework to study the resistance of quantum networks to noisy environments based on classical percolation in statistical mechanics. To make the analytical framework feasible, the following assumptions are made:

(1) Quantum nodes are spatially distributed according to network topology.

(2) Each node is able to send and receive an entangled quantum state at the same time.

(3) Initially, any two adjacent nodes share an entangled state (initial link) that is used to achieve one round of quantum communication.

(4) The presence or absence of an entangled state between two nodes is independent of the presence or absence of any other entangled state.

(5) Any two nodes in the quantum network can establish a new entangled state (end-to-end link) for communication through entanglement swapping.

According to previous assumptions, we can find that the quantum network always remains connected in an ideal environment. Namely, two arbitrary nodes can achieve a round of communication tasks with perfect entanglement swapping, unitary transformation and other operations. A more practical situation of a quantum network in a noisy environment is considered below.

Begin with randomly selecting an initial link (|φ〉) from the quantum network whose density matrix formalism is ρ=|φ〉〈φ|. The evolution of a state ρ under noisy quantum channels, where the Markovian approximation is valid, is governed by the Lindblad master equation [8,37],

(1) $$\frac{\partial \rho }{\partial t}=-i\left[H_{s},\rho \right]+\sum_{i,\alpha }\left(L_{i,\alpha }\rho L_{i,\alpha }^{†}-\frac{1}{2}\left\{L_{i,\alpha }^{†}L_{i,\alpha },\rho \right\}\right),$$

where the first term is the unitary evolution with Hamiltonian $H_{s}$, and the second term is the nonunitary contribution. To derive the Lindblad master equation, the Born and Markov approximations are used to determine the operator $L_{i,\alpha }$, where $L_{i,\alpha }=\sqrt{k_{i,\alpha }}\sigma_{\alpha }^{\left(i\right)}$ is the Lindblad operator acting on the *i*th qubit used to describe the coupling of the system to its environment. Each α corresponds to one type of quantum noise, and $\sigma_{\alpha }$ is a 2 × 2 matrix, and the constant $k_{i,\alpha }$ has the dimension of the inverse time. To solve the equation, one can make a change in variables to eliminate the first term. In this article, $H_{s}$ is considered independent with time. Without a loss of generality, we assume that $H_{s}=0$.

The evolved state ρ(t) can be obtained by solving Equation (1) with the condition that the initial quantum state and quantum noises are known.

To characterize the variation in the initial state, we can calculate the fidelity that indicates the degree of overlap between the initial and evolved states. It is

(2) $$\begin{array}{cc} F & =\langle \varphi |\rho (t)|\varphi \rangle . \end{array}$$

Obviously, the value of *F* decreases as the evolution time increases. *F* represents the probability of keeping consistent with the initial state in the evolution [38]. Hence, a perfect quantum channel between the nodes can keep the nodes connected with probability *F*. *F* can be regarded as the probability of edge occupation, which is equivalent to the probability of an edge being connected successfully in a classical network.

According to the bond percolation model, one can declare each edge (evolved state) in a noisy network to be in one of two states at time *t*: connected successfully with probability *F* and unconnected with probability 1−F. Therefore, there also exists a bond percolation threshold probability, $F_{c}$, such that a GCC can be established in quantum networks if and only if $F>F_{c}$.

Furthermore, $F_{c}$ determined a critical value of evolution time for the quantum network such that keeping stable is possible, called maximum time $t_{c}$. Considering a quantum network with an arbitrary pure state as the initial edge, the maximum time for the quantum network to remain stable under noise can be obtained by solving the following equation.

(3) $$\begin{array}{c} F=F_{c}. \end{array}$$

Equation (3) can be solved by setting the initial conditions (quantum state and quantum network topology) to get the threshold value $t_{c}$ of the quantum network under noise environments.

The analytical framework has applicability to different quantum network structures under kinds of noise environments. Notably, it applies to quantum networks from the initial state distribution to the beginning of quantum communication, which does not involve entanglement swapping. Hence, the maximum duration of a quantum network is affected by the initial state, the structure and the noise of a quantum network. The following sections illustrate how to use our framework to analyze quantum networks by specific examples.

### 3.2. Amplitude Damping and Phase Damping Noises

Amplitude damping and phase damping are two important examples of open quantum system evolution. Amplitude damping is the description of energy dissipation, while phase damping describes the loss of quantum information without a loss of energy [8]. Here, we assume a two-qubit entangled state shared by two nodes under amplitude damping noise and phase damping noise quantum channel, which are, respectively, described by the Lindblad operator $L_{i,-}$ and $L_{i,z}$. For simplicity, let $k_{i,-}=\tau_{i},k_{i,z}=\gamma_{i}.$ That is, we consider $\left(\left(L_{1,\alpha },L_{2,\alpha }\right),\;\alpha =-,z\right)$ noisy channel to solve the master Equation (1). Accordingly, the Lindblad master equation is of the form

(4) $$\begin{array}{cc} \frac{\partial \rho }{\partial t}= & \tau_{1}\left(\sigma_{-}^{\left(1\right)}\rho \sigma_{+}^{\left(1\right)}-\frac{1}{2}\sigma_{+}^{\left(1\right)}\sigma_{-}^{\left(1\right)}\rho -\frac{1}{2}\rho \sigma_{+}^{\left(1\right)}\sigma_{-}^{\left(1\right)}\right)\\ \; & +\tau_{2}\left(\sigma_{-}^{\left(2\right)}\rho \sigma_{+}^{\left(2\right)}-\frac{1}{2}\sigma_{+}^{\left(2\right)}\sigma_{-}^{\left(2\right)}\rho -\frac{1}{2}\rho \sigma_{+}^{\left(2\right)}\sigma_{-}^{\left(2\right)}\right)\\ \; & \gamma_{1}\left(\sigma_{z}^{\left(1\right)}\rho \sigma_{z}^{\left(1\right)}-\rho \right)+\gamma_{2}\left(\sigma_{z}^{\left(2\right)}\rho \sigma_{z}^{\left(2\right)}-\rho \right), \end{array}$$

where $\sigma_{-}=\left(\begin{array}{cc} 0 & 1\\ 0 & 0 \end{array}\right)$, $\sigma_{+}=\left(\begin{array}{cc} 0 & 0\\ 1 & 0 \end{array}\right)$ and $\sigma_{z}=\left(\begin{array}{cc} 1 & 0\\ 0 & -1 \end{array}\right)$.

The master equation reduces to four diagonal equations and six off-diagonal equations, which are

(5) $$\left\{\begin{array}{cc} \; & \frac{\partial \rho_{00}}{\partial t}=\tau_{1}\rho_{22}+\tau_{2}\rho_{11},\\ \; & \frac{\partial \rho_{11}}{\partial t}=\tau_{1}\rho_{33}-\tau_{2}\rho_{11},\\ \; & \frac{\partial \rho_{22}}{\partial t}=-\tau_{1}\rho_{22}+\tau_{2}\rho_{33},\\ \; & \frac{\partial \rho_{33}}{\partial t}=-\tau_{1}\rho_{33}-\tau_{2}\rho_{33}, \end{array}\right.$$

and

(6) $$\left\{\begin{array}{cc} \; & \frac{\partial \rho_{01}}{\partial t}=\tau_{1}\rho_{23}-\frac{1}{2}\tau_{2}\rho_{01}-2\gamma_{2}\rho_{01},\\ \; & \frac{\partial \rho_{02}}{\partial t}=-\frac{1}{2}\tau_{1}\rho_{02}+\tau_{2}\rho_{13}-2\gamma_{1}\rho_{02},\\ \; & \frac{\partial \rho_{03}}{\partial t}=-\frac{1}{2}\tau_{1}\rho_{03}-\frac{1}{2}\tau_{2}\rho_{03}-2\gamma_{1}\rho_{03}-2\gamma_{2}\rho_{03},\\ \; & \frac{\partial \rho_{12}}{\partial t}=-\frac{1}{2}\tau_{1}\rho_{12}-\frac{1}{2}\tau_{2}\rho_{12}-2\gamma_{1}\rho_{12}-2\gamma_{2}\rho_{12},\\ \; & \frac{\partial \rho_{13}}{\partial t}=-\frac{1}{2}\tau_{1}\rho_{13}-\tau_{2}\rho_{13}-2\gamma_{1}\rho_{13},\\ \; & \frac{\partial \rho_{23}}{\partial t}=-\tau_{1}\rho_{23}-\frac{1}{2}\tau_{2}\rho_{23}-2\gamma_{2}\rho_{23}. \end{array}\right.$$

We consider an arbitrary two-partite pure state |φ〉 as the initial state (t=0), which is expressed as

(7) $$\begin{array}{c} |\varphi \rangle =\lambda_{0}|00\rangle +\lambda_{1}|01\rangle +\lambda_{2}|10\rangle +\lambda_{3}|11\rangle \end{array}$$

where $\lambda_{0},\lambda_{1},\lambda_{2}$ and $\lambda_{3}$ are real and satisfy $|\lambda_{0}{|}^{2}+|\lambda_{1}{|}^{2}+|\lambda_{2}{|}^{2}+{\left|\lambda_{3}\right|}^{2}=1.$ Its density matrix is

(8) $$\begin{array}{c} \rho \left(0\right)=\left(\begin{array}{cccc} \lambda_{0}^{2} & \lambda_{0}\lambda_{1} & \lambda_{0}\lambda_{2} & \lambda_{0}\lambda_{3}\\ \lambda_{1}\lambda_{0} & \lambda_{1}^{2} & \lambda_{1}\lambda_{2} & \lambda_{1}\lambda_{3}\\ \lambda_{2}\lambda_{0} & \lambda_{2}\lambda_{1} & \lambda_{2}^{2} & \lambda_{2}\lambda_{3}\\ \lambda_{3}\lambda_{0} & \lambda_{3}\lambda_{1} & \lambda_{3}\lambda_{2} & \lambda_{3}^{2} \end{array}\right), \end{array}$$

that is, the boundary conditions for Equations (5) and (6) are, respectively, $\rho_{jj}\left(0\right)=\lambda_{j}^{2}$ and $\rho_{jk}\left(0\right)=\lambda_{j}\lambda_{k},$ where j<k and j,k=0,1,2,3. Therefore, the analytical form of an arbitrary two-partite pure state evolution under amplitude damping and phase damping noises is

(9) $$\rho \left(t\right)=\left(\begin{array}{cccc} \rho_{00}\left(t\right) & \rho_{01}\left(t\right) & \rho_{02}\left(t\right) & \rho_{03}\left(t\right)\\ \rho_{10}\left(t\right) & \rho_{11}\left(t\right) & \rho_{12}\left(t\right) & \rho_{13}\left(t\right)\\ \rho_{20}\left(t\right) & \rho_{21}\left(t\right) & \rho_{22}\left(t\right) & \rho_{23}\left(t\right)\\ \rho_{30}\left(t\right) & \rho_{31}\left(t\right) & \rho_{32}\left(t\right) & \rho_{33}\left(t\right) \end{array}\right),$$

where

(10) $$\left\{\begin{array}{cc} \rho_{00}\left(t\right)= & 1+\lambda_{3}^{2}e^{-t(\tau_{1}+\tau_{2})}-\left(\lambda_{1}^{2}+\lambda_{3}^{2}\right)e^{-t\tau_{2}}-\left(\lambda_{2}^{2}+\lambda_{3}^{2}\right)e^{-t\tau_{1}},\\ \rho_{11}\left(t\right)= & \left(\lambda_{1}^{2}+\lambda_{3}^{2}\right)e^{-t\tau_{2}}-\lambda_{3}^{2}e^{-t(\tau_{1}+\tau_{2})},\\ \rho_{22}\left(t\right)= & \left(\lambda_{2}^{2}+\lambda_{3}^{2}\right)e^{-t\tau_{1}}-\lambda_{3}^{2}e^{-t(\tau_{1}+\tau_{2})},\\ \rho_{33}\left(t\right)= & \lambda_{3}^{2}e^{-t(\tau_{1}+\tau_{2})}, \end{array}\right.$$

and

(11) $$\left\{\begin{array}{cc} \rho_{01}\left(t\right)\left(\rho_{10}\left(t\right)\right)= & \left(\lambda_{0}\lambda_{1}+\lambda_{2}\lambda_{3}\right)e^{-\frac{t}{2}\left(\tau_{2}+4\gamma_{2}\right)}-\lambda_{2}\lambda_{3}e^{-\frac{t}{2}\left(2\tau_{1}+\tau_{2}+4\gamma_{2}\right)},\\ \rho_{02}\left(t\right)\left(\rho_{20}\left(t\right)\right)= & \left(\lambda_{0}\lambda_{2}+\lambda_{1}\lambda_{3}\right)e^{-\frac{t}{2}\left(\tau_{1}+4\gamma_{1}\right)}-\lambda_{1}\lambda_{2}e^{-\frac{t}{2}\left(\tau_{1}+2\tau_{2}+4\gamma_{1}\right)},\\ \rho_{03}\left(t\right)\left(\rho_{30}\left(t\right)\right)= & \lambda_{0}\lambda_{3}e^{-\frac{t}{2}\left(\tau_{1}+\tau_{2}+4\gamma_{1}+4\gamma_{2}\right)},\\ \rho_{12}\left(t\right)\left(\rho_{21}\left(t\right)\right)= & \lambda_{1}\lambda_{2}e^{-\frac{t}{2}\left(\tau_{1}+\tau_{2}+4\gamma_{1}+4\gamma_{2}\right)},\\ \rho_{13}\left(t\right)\left(\rho_{31}\left(t\right)\right)= & \lambda_{1}\lambda_{3}e^{-\frac{t}{2}\left(\tau_{1}+2\tau_{2}+4\gamma_{1}\right)},\\ \rho_{23}\left(t\right)\left(\rho_{32}\left(t\right)\right)= & \lambda_{2}\lambda_{3}e^{-\frac{t}{2}\left(2\tau_{1}+\tau_{2}+4\gamma_{2}\right)}. \end{array}\right.$$

The fidelity is

(12) $$\begin{array}{cc} F & =\langle \varphi |\rho \left(t\right)|\varphi \rangle =\sum_{j,k=0}^{3}\lambda_{j}\lambda_{k}\rho_{jk}\left(t\right). \end{array}$$

By setting the initial conditions that the quantum state is an arbitrary two-partite pure state and the bond percolation threshold probabilities for the quantum network is $F_{c}$, one can solve Equation (13) to obtain the maximum time that the quantum network can be connected.

(13) $$\begin{array}{c} \sum_{j,k=0}^{3}\lambda_{j}\lambda_{k}\rho_{jk}\left(t\right)=F_{c}. \end{array}$$

It is well-known that an arbitrary two-qubit state can be written as a superposition of four Bell states. In another aspect, Bell states are always used to be the initial state of a quantum communication network [7,24]. Therefore, we consider the fidelity of one of these four Bell states $|\phi^{\pm }\rangle =\frac{1}{\sqrt{2}}(|00\rangle \pm \left|11\rangle \right)$ and $|\psi^{\pm }\rangle =\frac{1}{\sqrt{2}}(|01\rangle +\left|10\rangle \right)$ under amplitude damping and phase damping noises. Substituting the coefficients of $|\phi^{\pm }\rangle$ and $|\psi^{\pm }\rangle$ into Equation (12), the fidelity of the four states are, respectively,

(14) $$\begin{array}{c} F_{|\phi^{\pm }\rangle }=\frac{1}{4}\left(2+2e^{-t(\tau_{1}+\tau_{2})}+2e^{-\frac{t}{2}\left(\tau_{1}+\tau_{2}+4\gamma_{1}+4\gamma_{2}\right)}-e^{-t\tau_{1}}-e^{-t\tau_{2}}\right) \end{array}$$

and

(15) $$\begin{array}{c} F_{|\psi^{\pm }\rangle }=\frac{1}{4}\left(e^{-t\tau_{1}}+e^{-t\tau_{2}}+2e^{-\frac{t}{2}\left(\tau_{1}+\tau_{2}+4\gamma_{1}+4\gamma_{2}\right)}\right). \end{array}$$

**Figure 3:**
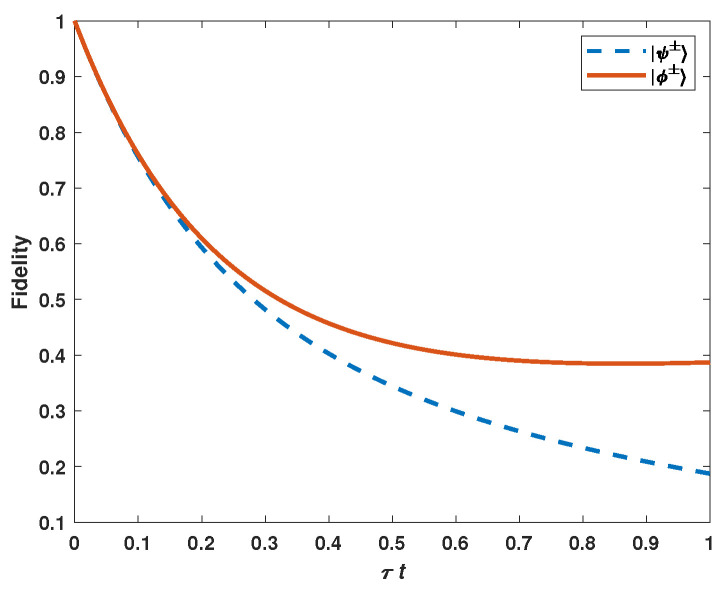
The fidelities of Bell states under amplitude damping and phase damping noises. For simulation, we consider the following parameters as identical: $\tau_{1}=\tau_{2}=\gamma_{1}=\gamma_{2}=\tau$.

Equations (14) and (15) indicate that the fidelity decreases with the increasing value of evolution time for a given initial quantum state (see Figure 3). The figure shows the effect of initial quantum state selection and evolution time on fidelity. It finds that states $|\phi^{\pm }\rangle$ show greater resistance to the two types of noise than states $|\psi^{\pm }\rangle$.

We use one of the Bell states as the initial link to further explain our analytical framework. By introducing examples of specific graphs, we illustrate how to calculate the critical value of time.

### 3.3. Regular Quantum Networks

To demonstrate the effectiveness of the analytical framework, we focus on the particular quantum networks used in entanglement-based QKD protocols [24]. Without loss of generality, we consider a quantum network whose neighboring nodes share a maximally entangled state $|\psi^{-}\rangle =\frac{1}{\sqrt{2}}(|01\rangle -\left|10\rangle \right)$, and the quantum network under two common noises, amplitude damping and phase damping noises.

**Figure 4:**
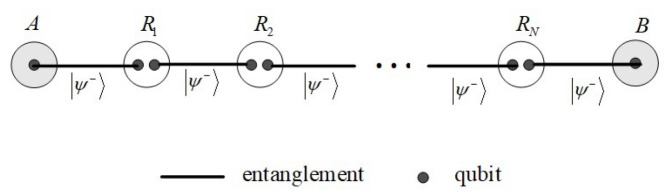
Quantum network in a one-dimensional chain.

First, we consider the situation of a one-dimensional chain that consists of two nodes connected by intermediate nodes, as shown in Figure 4. Obviously, communication parties (A and B) can choose the only path, $A\to R_{1}\to R_{2}\to \cdots \to R_{N}\to B$, to realize quantum key distribution by entanglement swapping. In order to ensure communication between the two sides, any two adjacent nodes in the path should be connected. Consistently with the standard bond percolation, it can be deduced that the stability time of this network is possible if and only if F=1. This is a very demanding condition for quantum communication in noisy environments, which is only satisfied when t=0.

**Figure 5:**
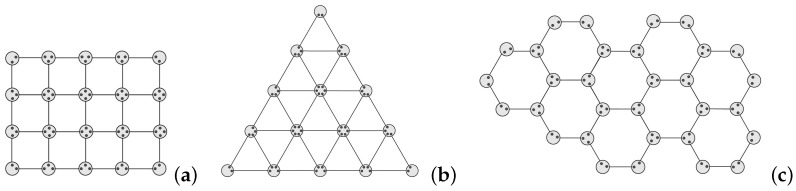
Quantum networks in two-dimensional lattices. (**a**) Square; (**b**) Triangle; (**c**) Honeycomb.

The above analysis can be generalized to two-dimensional lattices, such as squares, triangles and honeycombs (Figure 5). In quantum networks with a regular distribution of nodes, the nodes can choose different paths for communication. It is difficult to find all the paths and calculate the stability time. We give the critical condition of stability time based on bond percolation.

For the case of square lattices, we may assume that each quantum state associated with the edge is independently affected by the same noise described by Equation (4). Then, the quantum state evolves into a mixed state, which is consistent with the initial state with the probability of *F*. The fidelity of the initial state evolving to the final state is given by Equation (15). More specifically, each edge is occupied with probability *F* in the process of bond percolation.

According to the bond percolation threshold probabilities of the two-dimensional lattices listed in Table 1 [13], we can calculate the critical value of evolution time $t_{c}.$

To study the stability time of quantum networks further, we study the critical value of time associated with different experimental network models. We consider the two-qubit NMR system as in Ref. [39]. By solving the master equation in the Lindblad form, we can analyze the influence of the noise channels acting on the two-qubit NMR entangled states, as well as the critical value of the quantum network connection time when all the quantum states are independently affected by the same noise.

We consider a decoherence model wherein a nuclear spin is subject to two noise channels, namely, a phase-damping channel and an amplitude-damping channel. We solve the master Equation (4) with the initial state $|\psi^{-}\rangle$ state, together with the experimentally measured values of the spin-lattice relaxation rates for the two-spin homonuclear systems of BTC acid. According to the experimentally measured values of the system, $\tau_{1}=0.264$ s${}^{-1}$, $\tau_{2}=0.255$ s${}^{-1}$, $\gamma_{1}=3.741$ s${}^{-1}$, $\gamma_{2}=3.048$ s${}^{-1}$ [39], we obtain

(16) $$\begin{array}{c} F=\frac{1}{4}\left(e^{-0.2640t}+e^{-0.2550t}+2e^{-13.8375t}\right), \end{array}$$

from which we can calculate the maximum value of time that the quantum network stays connected according to the percolation threshold for a square. From Equations (3) and (16) and Table 1, we have the critical value of evolution time as 0.2117 s.

**Figure 6:**
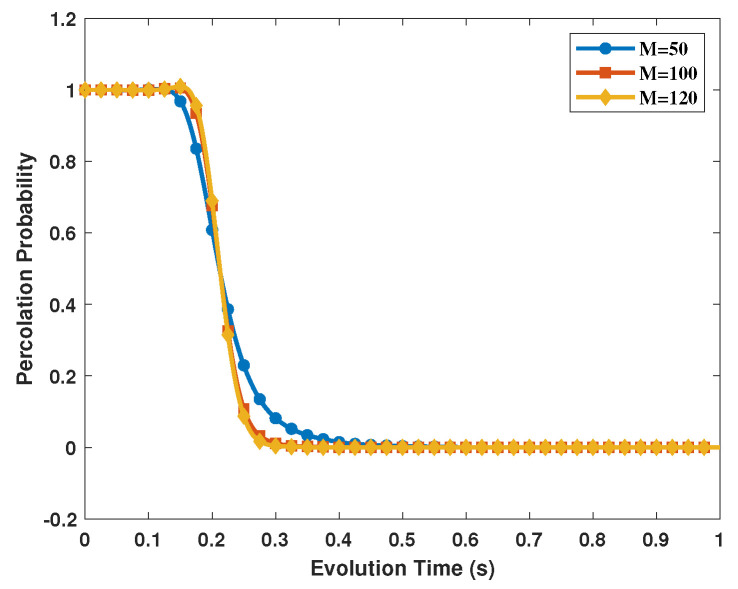
The distribution of the size of GCC for square quantum networks under amplitude damping and phase damping noises. The simulations were performed with N=M×M,M=50,100,120.

We have performed simulations of square networks to verify the above result (as shown in Figure 6). We use the Monte-Carlo simulation method to calculate the percolation probability, and the simulation tool is Matlab. During the simulation, we set the probability of a random occupied edge as *F*, which is determined by the evolution time *t* based on Equation (16). For each given *t*, we repeat the percolation process 1000 times to estimate the proportion of percolating times. Then, the distribution of the size of GCC can be obtained by changing *t* from 0 s to 1 s. The three curves show the distribution of the size of GCC in the network for N=M×M,M=50,100,120. We calculate the value of critical time is approximately 0.21 s, through this simulation. The calculated result and simulation result is in good agreement.

**Figure 7:**
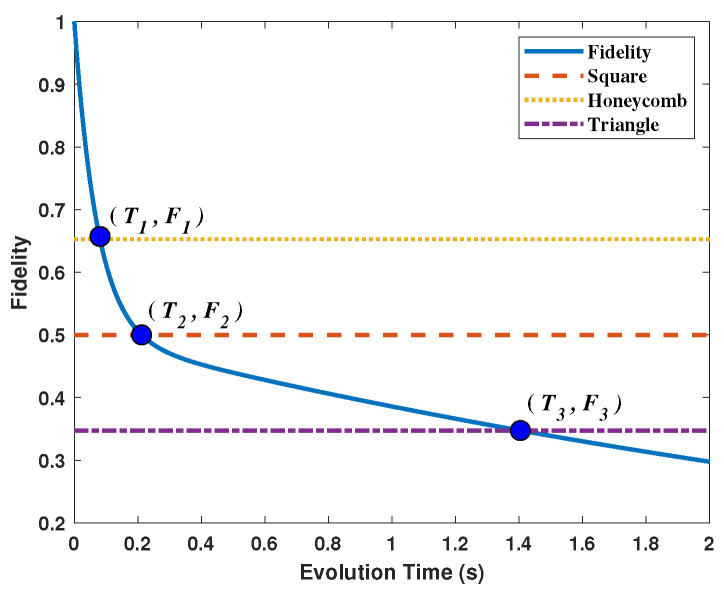
The critical times for triangle, square and honeycomb quantum networks. The three intersection points are $\left(F_{1},T_{1}\right)=\left(0.6527,0.0810\right)$, $\left(F_{2},T_{2}\right)=\left(0.5,0.2117\right)$ and $\left(F_{3},T_{3}\right)=\left(0.3473,1.4044\right)$, respectively.

Similarly, one can calculate the critical times of 1.4044 s and 0.0810 s for the cases of triangle and honeycomb lattices, respectively. In Figure 7, we show the relationship between the critical time and the topology of quantum networks. Due to the evolution of the quantum states, the duration time of the quantum network varies with the initial network structures. Obviously, when the quantum nodes are distributed in a triangle lattice, the duration of this quantum network is longer than the other two cases. If the nodes are distributed according to a honeycomb lattice, the quantum network lasts the shortest time compared with the triangle and square lattices. Based on the above analysis, we see that the duration of a quantum network is closely related to the quantum node distribution in the quantum network. The analyses above suggest that it is possible to make the quantum network more resistant to noise by modifying the structure of the quantum networks through entanglement swapping or other means.

### 3.4. Complex Quantum Networks

Now we study the threshold value of time in the scenario of general random graphs of arbitrary distribution degree. At first, we introduce the generating functions [14] to calculate the size of GCC of random graphs,

(17) $$G_{0}\left(x\right)=\Sigma_{k=0}^{\infty }P\left(k\right)x^{k}$$

and

(18) $$G_{1}\left(x\right)=\Sigma_{k=1}^{\infty }P_{1}\left(k\right)x^{k-1},$$

where P(k) is the probability that a randomly chosen node on the graph has degree *k*, and $P_{1}\left(k\right)$ is the probability of the node reached by randomly selecting an edge. We use the bond percolation model to analytically compute the critical value of time that the network could keep for communication.

**Figure 8:**
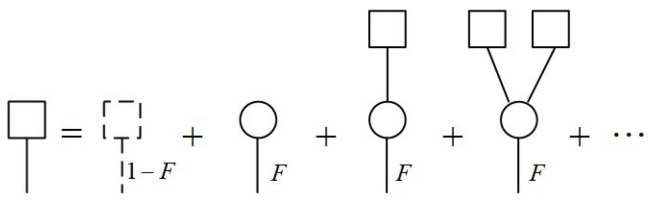
The graphical representation of the distribution of connected components generated by $H_{1}\left(x\right)$.

To start with, we randomly select an edge. Due to the evolution of the state corresponding to the edge in a noisy environment, the edge will keep the connection with probability *F* and be disconnected with probability 1−F. Simply, we suppose that the initial quantum state $|\psi^{-}\rangle$ is affected by amplitude damping and phase damping noises. The fidelity *F* is consistent with Equation (15). Consider the generating function $H_{1}\left(x\right)$ for the distribution of the sizes of components that are reached by choosing a random edge and following it to one of its ends. The distribution of connected components generated by $H_{1}\left(x\right)$ can be represented as in Figure 8.

$H_{1}\left(x\right)$ satisfies a self-consistency condition of the form

(19) $$H_{1}\left(x\right)=\left(1-F\right)+Fxq_{0}+Fxq_{1}H_{1}\left(x\right)+Fxq_{2}{\left(H_{1}\left(x\right)\right)}^{2}+\cdots ,$$

where $q_{k}$ is the probability that the quantum node has *k* edges besides the randomly selected edge. Hence, the generating function $H_{1}\left(x\right)$ is given by

(20) $$H_{1}\left(x\right)=1-F+FxG_{1}\left(H_{1}\left(x\right)\right),$$

where *F* is the probability that the edge preserves the initial link.

Similarly, the size of the components of the node we chose is the generating function $H_{0}\left(x\right)$,

(21) $$H_{0}\left(x\right)=xG_{0}\left(H_{1}\left(x\right)\right).$$

For a given *F*, denote $u=H_{1}\left(1\right)$, which is the probability of randomly choosing an edge to connect to a finite-connected component (FCC). We have

(22) $$u=1-F+FG_{1}\left(u\right).$$

Solving the above equation, one receives a critical value $F_{c}$. If $F<F_{c}$, there is a unique solution u=1, which implies that no GCC exists in the quantum networks. If $F\geq F_{c}$, we obtain a new solution u<1, which indicates the presence of GCC.

There are both FCC and GCC in a network. By selecting a node in the network randomly, the probability that the node can reach the GCC is regarded as part of the entire network that excludes the FCC. Set

(23) $$S=1-H_{0}\left(x\right).$$

The mean component size of a node arriving to FCC is given by

(24) $$<S>=H_{0}^{\prime }\left(x\right).$$

More specifically,

(25) $$<S>=H_{0}^{\prime }\left(1\right)=G_{0}\left(H_{1}\left(1\right)\right)+G_{0}^{\prime }\left(H_{1}\left(1\right)\right)H_{1}^{\prime }\left(1\right).$$

On the other hand, from Equation (20) we obtain

(26) $$H_{1}^{\prime }\left(1\right)=FG_{1}\left(H_{1}\left(1\right)\right)+FG_{1}^{\prime }\left(H_{1}\left(1\right)\right)H_{1}^{\prime }\left(1\right).$$

Clearly,

(27) $$H_{1}^{\prime }\left(1\right)=\frac{FG_{1}\left(H_{1}\left(1\right)\right)}{1-FG_{1}^{\prime }\left(H_{1}\left(1\right)\right)}.$$

Therefore, <S> becomes infinite at a critical value $F=F_{c}$ satisfying the equation $1-FG_{1}^{\prime }\left(H_{1}\left(1\right)\right)=0$, namely,

(28) $$F_{c}=\frac{1}{G_{1}^{\prime }\left(H_{1}\left(1\right)\right)}=\frac{1}{G_{1}^{\prime }\left(1\right)}.$$

Furthermore, we can calculate the maximal value of the time that the quantum network holds GCC by solving the following equation,

(29) $$\frac{1}{4}\left(e^{-t\tau_{1}}+e^{-t\tau_{2}}+2e^{-\frac{t}{2}\left(\tau_{1}+\tau_{2}+4\gamma_{1}+4\gamma_{2}\right)}\right)=\frac{1}{G_{1}^{\prime }\left(1\right)}.$$

Subsequently, we study the critical value of time that quantum networks stay connected in particularly complex network models. In Erdős-Rényi (ER) graphs, the distribution of degree follows a Poisson distribution in the large *N* limit. The probability p=z/N of the existence of an edge between any two nodes is the same, and $G_{0}\left(x\right)=G_{1}\left(x\right)=e^{z(x-1)}$, where *z* is the average degree of a node. Substituting the generating function of ER graphs into Equation (29), one has

(30) $$\frac{1}{4}\left(e^{-t\tau_{1}}+e^{-t\tau_{2}}+2e^{-\frac{t}{2}\left(\tau_{1}+\tau_{2}+4\gamma_{1}+4\gamma_{2}\right)}\right)=\frac{1}{z}.$$

**Figure 9:**
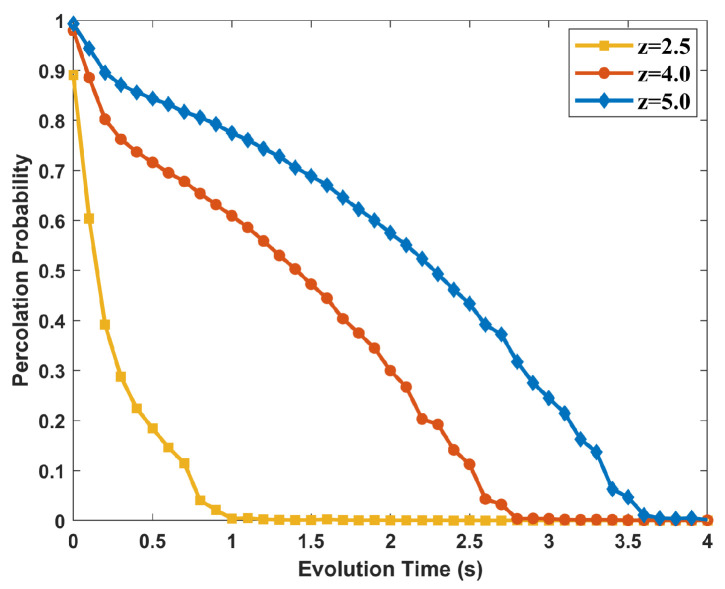
The distribution of the size of GCC for ER quantum networks under amplitude damping and phase damping noise.

We can calculate the critical time of the ER graphs with different average degrees by solving the above equation. For example, let z=2.5 and take $\tau_{1}=0.264$ s${}^{-1}$, $\tau_{2}=0.255$ s${}^{-1}$, $\gamma_{1}=3.741$ s${}^{-1}$, $\gamma_{2}=3.048$ s${}^{-1}$ [39], the critical time is calculated to be 0.86 s. It is easy to deduce that the larger the average degree *z* in the ER graphs, the longer time the quantum networks stay connected, which is consistent with intuitive imagination. Additionally, the numerical results show this conclusion as well. Let $\tau_{1}=0.264$ s${}^{-1}$, $\tau_{2}=0.255$ s${}^{-1}$, $\gamma_{1}=3.741$ s${}^{-1}$, $\gamma_{2}=3.048$ s${}^{-1}$ and the size of the ER networks used here is $N=10^{4}$. Setting the average degree *z* to be 2.5, 4.0 or 5.0, we set the occupation probability of each edge to be *F*, which is given by Equation (16). We vary the evolution time *t* from 0 s to 4 s and the distribution of the size of GCC from 0 to 1. The results of the percolation probability of quantum evolution time are shown in Figure 9, which is a verification of our calculation results from the analytical framework. It is obvious that the quantum network remains connected longer when z=5.0.

## 4. The Capacity of Quantum Networks

We have previously discussed the evolution of the initial link in a quantum network under noisy environments. In the remainder of this article, we discuss the capacity of the quantum network through the generation rate of end-to-end links.

In the classical domain, capacity is defined to analyze the ability to transport data in a network [40]. For a randomly distributed network with *N* nodes, the capacity can be defined as λ(n), in which each node transmits the total number of bits to its destination. Similarly, the capacity in a quantum network is defined as [41]

(31) $$Q_{th}=\lambda_{q}\left(n\right),$$

where $\lambda_{q}\left(n\right)$ denotes the number of end-to-end links generated between each pair of source and destination nodes. Due to the dynamics of a quantum network in a noisy environment, it is hard to calculate the total number of end-to-end links that can be generated between communication parties. We consider the important factors that affect the generation rate in the procedure of establishing end-to-end links. There are three important factors:

(1) Quantum noise. Real quantum systems suffer from unwanted interactions with the environment, i.e., each initial link (entangled state) evolved to be a mixed state independently.

(2) Routing algorithm. The choice of path determines the distance between two communication parties. Finding the optimal path in a quantum network is crucial.

(3) Entanglement swapping. The success probability of one entanglement swapping operation needs to be considered because the measurement procedure is inherently imperfect in practice.

Consider the simplest possible situation that (1), (2) and (3) are not considered; that is, the communication parties can always find the optimal path, and each intermediate node performs a perfect entanglement swapping to establish end-to-end links in an ideal quantum network without noise. The maximum number of end-to-end links between the source and the destination nodes is min$\left\{n_{s},n_{d}\right\}$, where $n_{s}$ and $n_{d}$ is the number of edges in the source node and destination node.

**Figure 10:**
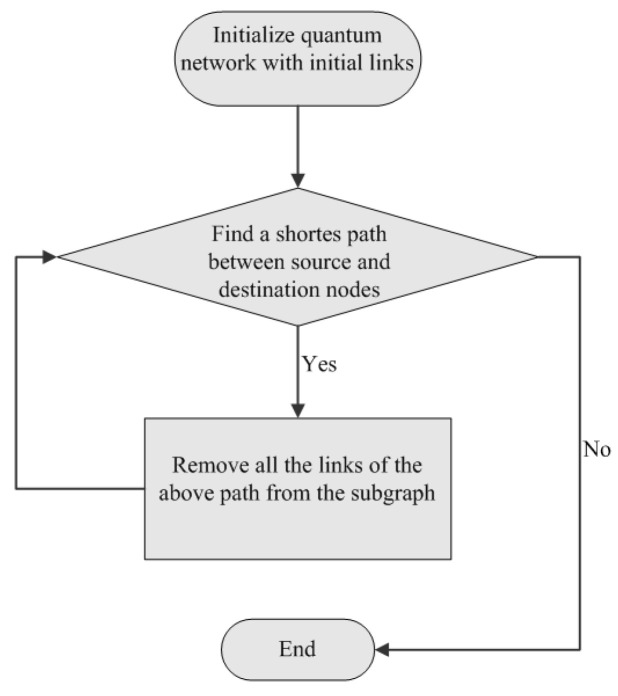
Flow chart of finding all the paths between the source and destination nodes by using a greedy algorithm.

We turn now to the situation that the initial link is affected by quantum noise, which is described by Equation (1). Moreover, we use the greedy algorithm to find the largest number of paths between source and destination nodes (see Figure 10). In the beginning, a greedy algorithm tries to find the shortest path between the source and destination nodes. If no connected path between communication parties exists, no end-to-end link is generated in this round of communication. If the shortest path is found, then remove all the links of the above path from the subgraph. Repeat the process until all paths connecting communication parties are found.

Let $l_{i},i=1,2,\cdots ,m$ is the length of the shortest path found at the i-th time, then $l_{1}\leq \cdots \leq l_{m}$ and m≤ min$\left\{n_{s},n_{d}\right\}$. If the success probability of one entanglement swapping operation is $P_{M}$, and it is identical among quantum nodes, we have the generation rate of an end-to-end link as

(32) $$R_{i}=F^{l_{i}}P_{M}^{l_{i}-1},$$

F is the fidelity that can be calculated by Equation (2). The establishing rate of end-to-end links between communication parties is

(33) $$\begin{array}{cc} R & =\sum_{i}^{m}R_{i}. \end{array}$$

Using $l_{1}\leq \cdots \leq l_{m}$ to obtain the inequality of capacity of the quantum network under noise. It is

(34) $$\begin{array}{cc} R & \leq mF^{l_{1}}P_{M}^{l_{1}-1}. \end{array}$$

Obviously, the capacity of a quantum network is related to the quantum noise, the topology of the quantum network and the successful probability of quantum measurement. The generation rate of end-to-end links falls off exponentially with the distance between two communication parties. Specifically, if the measurement procedure is perfect at each node, the generation rate is independent of the distance between the two communication parties when $t<t_{c}$. Compared to the former comparison results of specific examples under amplitude damping and phase damping noises, it is not hard to find that the conclusions, in this case, are consistent. For a regular quantum network, the capacity of the triangle is bigger than those of the square and honeycomb. For the ER quantum network, the capacity also increases as *z* increases.

## 5. Conclusions

We have studied the resistance of quantum networks to noisy environments via the proposed framework. By linking the fidelity with the percolation threshold, the maximum time for the quantum network to remain stable in noisy environments has been calculated under different network structures. For regular quantum networks, we found that the values of critical time can be exactly computed since the bond percolation threshold of a square, triangle and honeycomb can be solved exactly in the classic network. The results show that the quantum network of a triangle lattice maintains a longer communication capacity than that of square and honeycomb lattices in noisy environments. Additionally, we have given the general result for complex quantum networks and obtained the critical value of time for ER quantum networks under the phase damping and amplitude damping noises. We found that the duration time of ER quantum networks increases with the average degree of a node increasing. The calculation results obtained from our framework are in complete agreement with the numerical results as well. Moreover, we found that the capacity of a quantum network is independent of the communication distance when the measurement is perfect and the evolution time $t<t_{c}$.

The proposed framework provides a different perspective on the dynamics of quantum networks in noisy environments. It can help us calculate the maximum time for a given quantum network to remain stable and remind us to complete one round of quantum communication through noisy quantum networks in a limited time to ensure communication efficiency. The results of the analytical framework are also helpful in determining the design of quantum networks. Furthermore, based on the value of critical time, the time interval of each round of entanglement distribution can be controlled to maximize the use of quantum resources. Therefore, our framework may highlight the analysis and design of large-scale quantum communication networks.

## Figures and Tables

**Table 1:** The bond percolation threshold probabilities for various lattices in various dimensions.

Lattice	Bond Percolation
1d-Chain	1
2d-Honeycomb	1 − 2sin(π/18) ≈ 0.6527
2d-Square	0.5
2d-Triangle	2sin(π/18) ≈ 0.3473

## Data Availability

Not applicable.

## Abbreviations

The following abbreviations are used in this manuscript:

QKD	Quantum Key Distribution
GCC	Giant Connected Component
FCC	Finite Connected Component
NMR	Nuclear Magnetic Resonance
